# Adsorption and Purification of Baicalin from *Scutellaria baicalensis* Georgi Extract by Ionic Liquids (ILs) Grafted Silica

**DOI:** 10.3390/molecules26082322

**Published:** 2021-04-16

**Authors:** Yunchang Fan, Di Wu, Sheli Zhang

**Affiliations:** 1 College of Chemistry and Chemical Engineering, Henan Polytechnic University, Jiaozuo 454003, China; 211812010008@home.hpu.edu.cn; 2 College of Science and Technology, Jiaozuo Teachers College, Jiaozuo 454000, China; 1295006035@jzsz.edu.cn

**Keywords:** ionic liquids (ILs), grafted silica, adsorption, baicalin, purification

## Abstract

Baicalin which has multiple biological activities is the main active component of the root of *Scutellaria baicalensis* Georgi (SBG). Although its isolation and purification by adsorption methods have aroused much interest of the scientific community, it suffered from the poor selectivity of the adsorbents. In this work, an environmentally benign method was developed to prepare ionic liquids (ILs) grafted silica by using IL 1-butyl-3-methylimidazolium bis(trifluoromethylsulfonyl)imide ([C_4_mim]NTf_2_) and ethanol as reaction media. The IL 1-propyl-3-methylimidazolium chloride ([C_3_mim]Cl) grafted silica ([C_3_mim]^+^Cl^−^@SiO_2_) was used to adsorb and purify baicalin from the root extract of *Scutellaria baicalensis* Georgi (SBG). Experimental results indicated that the adsorption equilibrium can be quickly achieved (within 10 min). The adsorption behavior of [C_3_mim]^+^Cl^−^@SiO_2_ for baicalin was in good agreement with Langmuir and Freundlich models and the adsorption was a physisorption process as suggested by Dubinin–Radushkevich model. Compared with commercial resins, [C_3_mim]^+^Cl^−^@SiO_2_ showed the strongest adsorption ability and highest selectivity. After desorption and crystallization, a purity of baicalin as high as 96.5% could be obtained. These results indicated that the ILs grafted silica materials were promising adsorbents for the adsorption and purification of baicalin and showed huge potential in the purification of other bioactive compounds from natural sources.

## 1. Introduction

*Scutellaria baicalensis* Georgi (SBG) is one of the traditional medicine plants in Asia. Its roots are widely used as a remedy for the treatment of infection of the respiratory tract, inflammation, fever, and have anticancer, antimutagen, antiradical and lowering blood pressure effects [1,2,3]. Furthermore, the roots of SBG are also regarded as a popular functional food and usually used to stew soup with pork tripe or chicken in Asia [4]. Recent work reveals that baicalin is the main active ingredient of the roots of SBG and exhibits high antioxidant and hydroxyl radicals scavenging activity, which makes baicalin a potential cancer-chemopreventive agent against tumor promotion [5,6]. The extraction and purification of baicalin from SBG have aroused extensive concern among the scientific community. At present, many extraction methods including ultrasound-assisted extraction (UAE) [7,8], supercritical fluid extraction (SFE) [9], ultrahigh pressure extraction (UPE) [10], microwave-assisted extraction (MAE) [11], and heat reflux extraction (HRE) [7,12,13,14,15] were developed to isolate baicalin from SBG. After extraction, a purification procedure is needed to obtain high purity baicalin. In this context, adsorption method with polyamide [13] or macroporous resin [15] as adsorbents and counter-current chromatography (CCC) [8,14] have been used to purify baicalin from the extract of SBG. Although CCC was successively applied to purify baicalin, this technique involves the use of sophisticated instruments, which increases the operating cost [15]. Adsorption is a low-cost method with the advantage of ease of operation. Du et al. [15] used a non-polar macroporous resin (model HPD-100) to separate and purify baicalin from the extract of SBG. This resin exhibited good adsorption and desorption properties. After treatment with HPD-100 resin, the purity of baicalin was 58.3% with a recovery yield of 80.4%. Chi et al. [13] used polyamide resin as an adsorbent to purify baicalin and studied the adsorption mechanism. The experimental results showed that the adsorption behavior of polyamide to baicalin agreed with the Freundlich equation and the baicalin with a purity of 33.86% (wt%) was obtained after treatment with polyamide.

**Figure 1:**
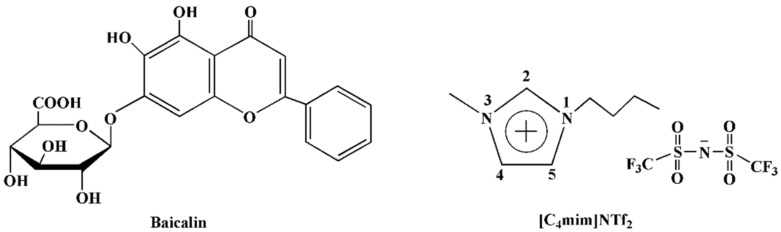
Chemical structures of baicalin and 1-butyl-3-methylimidazolium bis(trifluoromethylsulfonyl)imide ([C_4_mim]NTf_2_).

**Figure 2:**
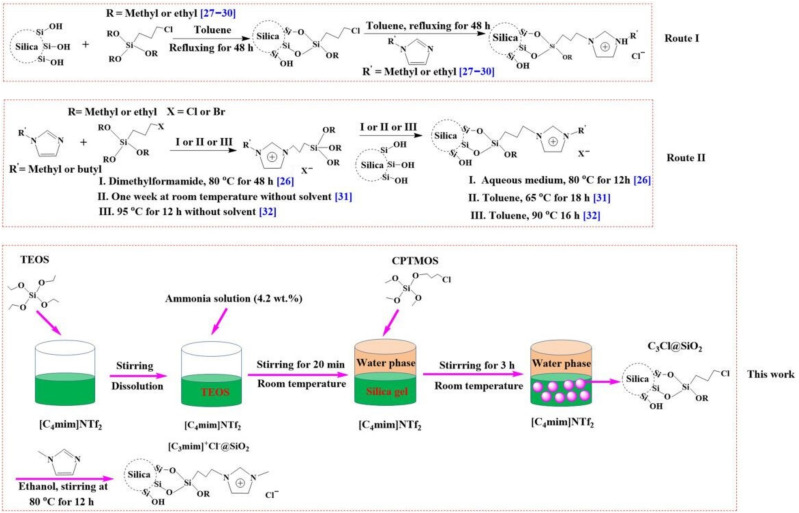
Schematic diagrams of the preparation of ILs grafted silica by reported routes and the proposed method.

Based on the above discussion, there is an urgent necessity to develop a high-effective absorbent in order to obtain high-pure baicalin. As shown in Figure 1, baicalin is an organic acid, has a large π-conjugated structure and contains hydroxyl groups. These characteristics mean that baicalin has the tendency to form hydrogen bonds and to interact with other π-conjugated systems via π-π stacking. Therefore, the adsorbent which has a π-conjugated structure and contains the groups easy to form hydrogen bonds may be a good choice to achieve high effective and selective adsorption for baicalin. Recently, ionic liquids (ILs) and their analogues, deep eutectic solvents (DESs) have been widely applied to extract bioactive compounds due to their excellent solubility for organic compounds [16,17,18,19,20,21]. Furthermore, imidazolium-based ILs have a π-conjugated structure and the H atom of C2–H on the imidazolium cation has the tendency to form hydrogen bonds with strongly electronegative atoms (e.g., O, N, and halide) (Figure 1) [22,23,24,25]. Therefore, the absorbents with their surfaces modified by ILs may provide high adsorption capacity and selectivity. At present, silica is usually used as carrier for immobilizing ILs due to its low-cost and nontoxic properties [26,27]. Two routes are usually adopted to covalently graft ILs to the surface of silica (Figure 2): (I) halide-containing group (e.g., 3-chloropropyl) was firstly introduced on the surface of silica via the reaction between silane coupling agents (e.g., (3-chloropropyl)trimethyoxysilane (CPTMOS)) and silica. Subsequently, the ILs were immobilized on the surface of silica through quaternization reaction [28,29,30,31]. (II) The ILs containing halide group were firstly synthesized via the reaction between alkylimidazole and silane coupling agents, following the grafting ILs to the silica surface via alcohol condensation [27,32,33]. As shown in Figure 2, current methods for the preparation of IL modified silica involve the use of dimethylformamide and toluene as reaction media. It is well known that dimethylformamide is carcinogenic [34] and clinical studies suggest that toluene is a neurotoxin which causes cerebral white matter damage [35]. It is of great importance to explore an environmental benign method which avoids the use of high toxic solvents. Toxic experiments indicate that the IL, 1-butyl-3-methylimidazolium bis(trifluoromethylsulfonyl)imide ([C_4_mim]NTf_2_) is practically nontoxic towards marine and freshwater fish based on acute toxicity rating scale by fish and wildlife service (FWS) [36]. Additionally, the toxic assay by means of glucose-uptake inhibition experiments also shows that [C_4_mim]NTf_2_ is not toxic at a concentration of 5% (*v*/*v*) [37]. These observations suggest that [C_4_mim]NTf_2_ is a biocompatible solvent. Furthermore, [C_4_mim]NTf_2_ has good solubility to silane reagents, such as tetraethyl orthosilicate (TEOS) and CPTMOS.

Based on the above discussion, the present work suggested an environmentally benign method to prepare IL-supported silica (Figure 2): the silane reagents, TEOS and CPTMOS, were dissolved into [C_4_mim]NTf_2_ and the hydrolysis occurred at the interface between IL and water phases (TEOS was allowed to hydrolyze for 20 min before the addition of CPTMOS due to their different hydrolytic rates). The resultant product was reacted with *N*-methylimidazole in the medium of ethanol at 80 °C for 8 h to produce the IL-modified silica. The adsorption and desorption behavior of this material for baicalin was systematically studied. Finally, it is believed that the IL-grafted silica will have great potential in practical purification of baicalin and the adsorption and purification of other natural bioactive compounds.

## 2. Results and Discussion

### 2.1. Optimization of the Preparation Conditions of the ILs Grafted Silica

**Figure 3:**
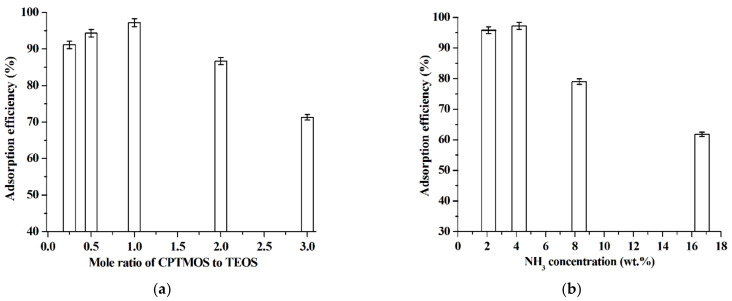
Effect of the mole ratio of CPTMOS to TEOS (**a**) (dosage of [C_4_mim]NTf_2_, 1.0 g; NH_3_ concentration, 4.2 wt%; quaternization reaction time, 12 h; mole ratio of MIM to C_3_Cl@SiO_2_ of the quaternization reaction, 3.0; adsorption test: [C_3_mim]^+^Cl^−^@SiO_2_, 40 mg; baicalin aqueous solution, 5.0 × 10^−5^ mol·L^−1^, pH 5.0, 10 mL; adsorption time, 1 h; adsorption temperature, 25 °C) and NH_3_ concentration (**b**) (dosage of [C_4_mim]NTf_2_, 1.0 g; mole ratio of CPTMOS to TEOS, 1.0; quaternization reaction time, 12 h; mole ratio of MIM to C_3_Cl@SiO_2_ of the quaternization reaction, 3.0; adsorption test: [C_3_mim]^+^Cl^−^@SiO_2_, 40 mg; baicalin aqueous solution, 5.0 × 10^−5^ mol·L^−1^, pH 5.0, 10 mL; adsorption time, 1 h; adsorption temperature, 25 °C) on the adsorption efficiency of [C_3_mim]^+^Cl^−^@SiO_2_. Experiments were conducted in triplicate.

In this work, the ILs grafted silica was prepared by two-step reactions: (I) the co-hydrolysis and co-condensation of CPTMOS and TEOS in [C_4_mim]NTf_2_ medium in the presence of ammonia solution (NH_3_) to produce C_3_Cl@SiO_2_. (II) The quaternization reaction between C_3_Cl@SiO_2_ and imidazole derivatives (MIM, C_2_OHIM, or BzIM). Therefore, the reaction conditions such as the mole ratio of CPTMOS to TEOS, the dosages of NH_3_, the reaction time of quaternization and the dosage of [C_4_mim]NTf_2_ were optimized using the preparation of [C_3_mim]^+^Cl^−^@SiO_2_ as a representative. As shown in Figure 3a, with the mole ratio of CPTMOS to TEOS increasing from 0.25 to 1.0, the adsorption ability of the final product [C_3_mim]^+^Cl^−^@SiO_2_ for baicalin increases. The reason lies in that CPTMOS is the active component, which can react with MIM to produce IL. However, further increase in the mole ratio of CPTMOS to TEOS leads to the decrease of the adsorption ability of [C_3_mim]^+^Cl^−^@SiO_2_. Primary experiments suggest that without the addition of TEOS, i.e., only using CPTMOS as starting material, the final product [C_3_mim]^+^Cl^−^@SiO_2_ is completely soluble in water. That is to say, high mole ratio of CPTMOS to TEOS results in the loss of IL from the surface of silica, subsequently decreasing the adsorption ability of [C_3_mim]^+^Cl^−^@SiO_2_. based on this observation, 1.0 is selected as the optimal mole ratio of CPTMOS to TEOS for the preparation of [C_3_mim]^+^Cl^−^@SiO_2_.

Generally, NH_3_ acts as catalyst in the hydrolysis and condensation of TEOS and CPTMOS [38,39]. The effect of NH_3_ concentration on the adsorption ability of [C_3_mim]^+^Cl^−^@SiO_2_ is shown in Figure 3b. As can be seen, [C_3_mim]^+^Cl^–^@SiO_2_ has stronger adsorption ability for baicalin at lower NH_3_ concentration. The reason may lie in that lower NH_3_ concentration means lower reaction rates, which results in the better co-condensation of TEOS and CPTMOS. Furthermore, when 2.1 wt% of NH_3_ is adopted, the hydrolysis rate of TEOS is slow (the generation of silica gel requires about 40 min). Therefore, 4.2 wt% of NH_3_ is selected for the following experiments.

**Figure 4:**
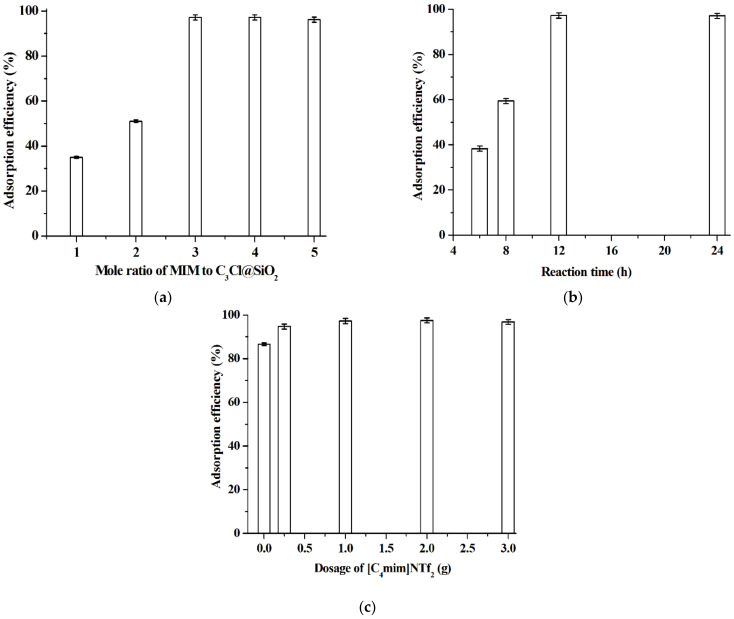
Effect of the mole ratio of MIM to C_3_Cl@SiO_2_ (**a**) (dosage of [C_4_mim]NTf_2_, 1.0 g; mole ratio of CPTMOS to TEOS, 1.0; NH_3_ concentration, 4.2 wt%; quaternization reaction time, 12 h; adsorption test: [C_3_mim]^+^Cl^−^@SiO_2_, 40 mg; baicalin aqueous solution, 5.0 × 10^−5^ mol·L^−1^, pH 5.0, 10 mL; adsorption time, 1 h; adsorption temperature, 25 °C), reaction time of quaternization (**b**) (dosage of [C_4_mim]NTf_2_, 1.0 g; mole ratio of CPTMOS to TEOS, 1.0; NH_3_ concentration, 4.2 wt%; mole ratio of MIM to C_3_Cl@SiO_2_ of the quaternization reaction, 3.0; adsorption test: [C_3_mim]^+^Cl^−^@SiO_2_, 40 mg; baicalin aqueous solution, 5.0 × 10^−5^ mol L^−1^, pH 5.0, 10 mL; adsorption time, 1 h; adsorption temperature, 25 °C) and dosage of [C_4_mim]NTf_2_ (**c**) (mole ratio of CPTMOS to TEOS, 1.0; NH_3_ concentration, 4.2 wt%; mole ratio of MIM to C_3_Cl@SiO_2_ of the quaternization reaction, 3.0; quaternization reaction time, 12 h; adsorption test: [C_3_mim]^+^Cl^−^@SiO_2_, 40 mg; baicalin aqueous solution, 5.0 × 10^−5^ mol·L^−1^, pH 5.0, 10 mL; adsorption time, 1 h; adsorption temperature, 25 °C) on the adsorption efficiency of [C_3_mim]^+^Cl^−^@SiO_2_. Experiments were conducted in triplicate.

To optimize the reaction conditions of quaternization, the mole ratio of MIM to C_3_Cl@SiO_2_ was firstly investigated and the results shown in Figure 4a indicate that excess amount of MIM is needed in order to convert more C_3_Cl@SiO_2_ to [C_3_mim]^+^Cl^−^@SiO_2_. Based on this observation, 3.0 is selected as the optimal mole ratio of MIM to C_3_Cl@SiO_2_. The effect of reaction time of quaternization shown in Figure 4b suggests that 12 h is enough for the quaternization reaction between MIM and C_3_Cl@SiO_2_. As shown in Figure 4c, with the use of [C_4_mim]NTf_2_ as reaction medium of the hydrolysis and condensation of TEOS and CPTMOS, [C_3_mim]^+^Cl^−^@SiO_2_ shows stronger adsorption ability for baicalin. The reason may lie in that the hydrolysis and condensation of TEOS and CPTMOS occur at the interface of [C_4_mim]NTf_2_ and water phases and thus the reaction rates are lower compared with the reaction system without the use of [C_4_mim]NTf_2_. Lower reaction rate means better co-condensation of TEOS and CPTMOS. Based on these results, 1.0 g of [C_4_mim]NTf_2_ is selected as the optimal IL dosage.

In summary, the optimal conditions for the preparation of the ILs grafted silica are as follows: mole ratio of CPTMOS to TEOS, 1.0; NH_3_ concentration, 4.2 wt%; mole ratio of MIM to C_3_Cl@SiO_2_, 3.0; reaction time, 12 h; dosage of [C_4_mim]NTf_2_, 1.0 g.

The *N*-(2-hydroxyethyl)imidazolium- and *N*-benzylimidazolium-based ILs grafted silica [C_3_C_2_OHim]^+^Cl^−^@SiO_2_ and [C_3_Bzim]^+^Cl^−^@SiO_2_ were also synthesized according to the above reaction conditions.

### 2.2. Characterization of ILs Grafted Silica

**Figure 5:**
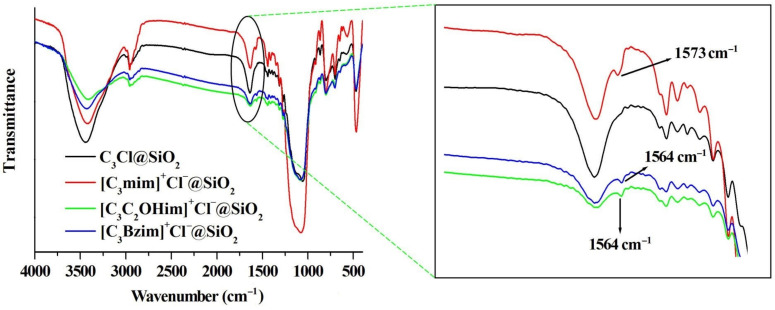
FT-IR spectra of C_3_Cl@SiO_2_, [C_3_mim]^+^Cl^−^@SiO_2_, [C_3_C_2_OHim]^+^Cl^−^@SiO_2_, and [C_3_Bzim]^+^Cl^−^@SiO_2_.

Elemental analysis and FT-IR spectra were used to confirm whether the ILs were grafted onto the surface of silica. Elemental analysis indicates that the N contents (wt%) of C_3_Cl@SiO_2_, [C_3_mim]^+^Cl^−^@SiO_2_, [C_3_C_2_OHim]^+^Cl^−^@SiO_2_ and [C_3_Bzim]^+^Cl^−^@SiO_2_ are <0.01%, 1.52%, 1.26%, and 1.26%, respectively. The increase in the N content confirms the successful grafting ILs onto the silica surface. The FT-IR spectra of C_3_Cl@SiO_2_, [C_3_mim]^+^Cl^−^@SiO_2_, [C_3_C_2_OHim]^+^Cl^−^@SiO_2_ and [C_3_Bzim]^+^Cl^−^@SiO_2_ are illustrated in Figure 5. As can be seen, compared with C_3_Cl@SiO_2_, a new absorption peak can be observed for the ILs grafted silica (1564^−1^ for [C_3_C_2_OHim]^+^Cl^−^@SiO_2_ and [C_3_Bzim]^+^Cl^−^@SiO_2_ and 1573 cm^−1^ for [C_3_mim]^+^Cl^−^@SiO_2_), which corresponds to the C=N stretching vibration of the imidazolium ring [40,41]. This observation also suggests that the ILs are successfully grafted onto the silica surface.

**Figure 6:**
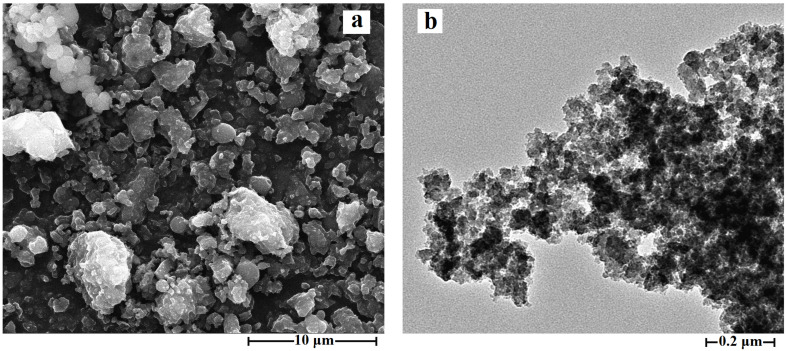
FE-SEM (**a**) and TEM (**b**) images of [C_3_mim]^+^Cl^−^@SiO_2_.

**Figure 7:**
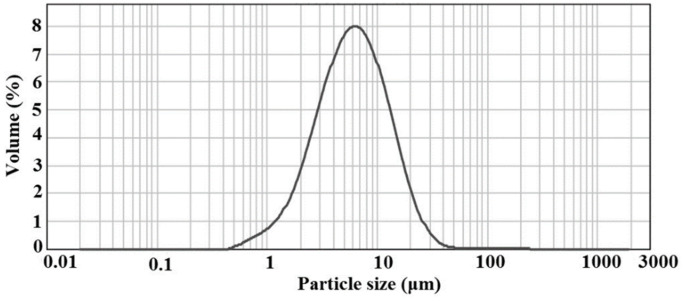
Particle size distribution of [C_3_mim]^+^Cl^−^@SiO_2_.

The TEM and SEM images of [C_3_mim]^+^Cl^−^@SiO_2_ are shown in Figure 6 and the TEM and SEM images of [C_3_C_2_OHim]^+^Cl^−^@SiO_2_ and [C_3_Bzim]^+^Cl^−^@SiO_2_ are demonstrated in Figures S1 and S2 in the Supplementary Materials. As can be seen from the TEM images, the ILs grafted silica products show a rough and wormhole-like structure. Their specific surface areas were determined and the experimental results indicate that the specific surface areas of [C_3_mim]^+^Cl^−^@SiO_2_, [C_3_C_2_OHim]^+^Cl^−^@SiO_2_ and [C_3_Bzim]^+^Cl^−^@SiO_2_ are 16.5 m^2^·g^−1^, 23.3 m^2^·g^−1^ and 18.4 m^2^·g^−1^, respectively. The SEM images of the ILs grated silica products (Figure 6 and Figures S1 and S2) show that all the products are amorphous and accumulations of small particles at micron scale. To further determine the particle size, all the products were tested by laser particle size analyzer and the results shown in Figure 7 and Figures S3 and S4 in the Supplementary Materials indicate that the average particle sizes of [C_3_mim]^+^Cl^−^@SiO_2_, [C_3_C_2_OHim]^+^Cl^−^@SiO_2_, and [C_3_Bzim]^+^Cl^−^@SiO_2_ are 5.9 μm, 6.8 μm, and 10.7 μm, respectively.

### 2.3. Adsorption Performance

**Figure 8:**
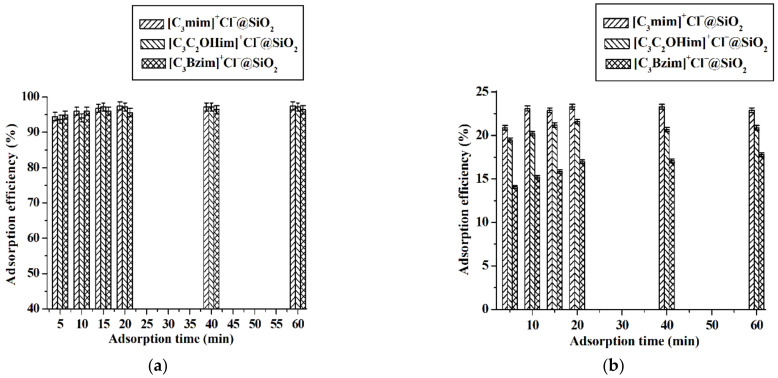
Adsorption efficiency of [C_3_mim]^+^Cl^−^@SiO_2_, [C_3_C_2_OHim]^+^Cl^−^@SiO_2_ and [C_3_Bzim]^+^Cl^−^@SiO_2_. (**a**) C_baicalin_ = 5.0 × 10^−5^ mol·L^−1^ and (**b**) C_baicalin_ = 9.0 × 10^−3^ mol·L^−1^. Experimental conditions: adsorbent, 40 mg; volume of baicalin aqueous solution, 10 mL; pH 5.0; adsorption temperature, 25 °C. Experiments were conducted in triplicate.

In this work, the three ILs grafted silica products were used to adsorb baicalin from aqueous phase and the results shown in Figure 8 suggest that at lower baicalin concentration (5.0 × 10^−5^ mol·L^−1^), the three ILs grafted silica particles have similar adsorption ability (>97%) and [C_3_mim]^+^Cl^−^@SiO_2_ has the strongest absorption ability compared with [C_3_C_2_OHim]^+^Cl^−^@SiO_2_ and [C_3_Bzim]^+^Cl^−^@SiO_2_ at higher baicalin concentration (9.0 × 10^−3^ mol·L^−1^). The reason may be that compared with [C_3_mim]^+^Cl^−^@SiO_2_, [C_3_C_2_OHim]^+^Cl^−^@SiO_2_, and [C_3_Bzim]^+^Cl^−^@SiO_2_ bear larger size groups (hydroxyethyl for [C_3_C_2_OHim]^+^Cl^−^@SiO_2_ and benzyl for [C_3_Bzim]^+^Cl^−^@SiO_2_), which hinders the interaction between adsorbents and baicalin decreasing the adsorption ability of adsorbents. This phenomenon is the so-called steric hindrance effect. To investigate the adsorption ability and mechanism of the ILs grafted silica particles, the equilibrium data were firstly fitted by Langmuir (Equation (3)) and Freundlich (Equation (4)) models and the results are listed in Table 1. As can be seen from Table 1, the 1/*n* values in the Freundlich equation are less than 1.0 (0.2959 to 0.3679), suggesting that the adsorption of baicalin on the ILs grafted silica can occur easily [15,42,43]. Besides, both of the two models are suitable to describe the adsorption behavior of ILs grafted silica for baicalin, indicating the fact that both monolayer and heterogeneous surface conditions exist under the selected experimental conditions. This phenomenon was also observed when macroporous resin was used to adsorb and purify baicalin [15]. To further explore the types of adsorption, the equilibrium data were analyzed with the Dubinin–Radushkevich isotherm model. The results listed in Table 1 indicate that the *E* values (average free energy) are in the range of 2.26 kJ·mol^−1^ to 2.44 kJ·mol^−1^, suggesting that the adsorption of baicalin onto the ILs grafted silica is a physisorption process due to *E* < 8.00 kJ·mol^−1^ [44,45].

Finally, considering the fact that [C_3_mim]^+^Cl^−^@SiO_2_ has the strongest adsorption ability for baicalin, it is selected in the following experiments.

### 2.4. Selection of the Adsorption Conditions of [C_3_mim]^+^Cl^−^@SiO_2_

**Figure 9:**
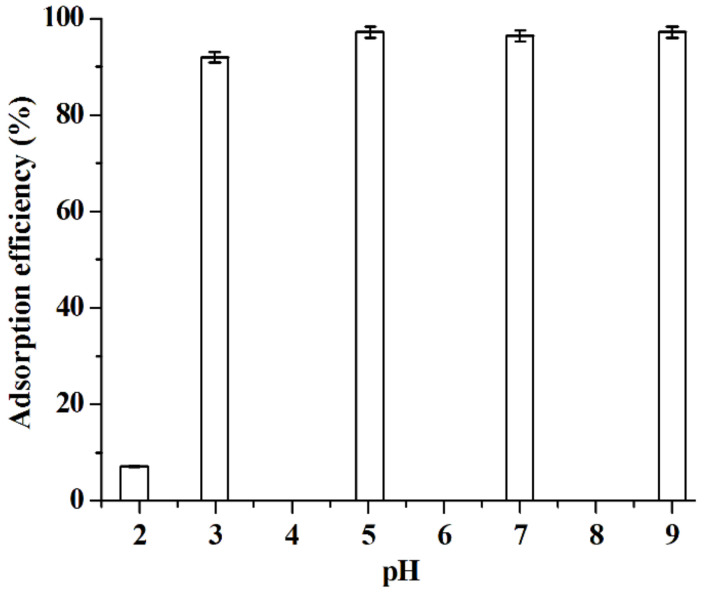
Effect of pH of water phase on the adsorption efficiency of [C_3_mim]^+^Cl^−^@SiO_2_. Experimental conditions: *C*_baicalin_ = 5.0 × 10^−5^ mol·L^−1^; [C_3_mim]^+^Cl^−^@SiO_2_, 40 mg; volume of baicalin aqueous solutable 10 mL; adsorption time, 10 min; adsorption temperature, 25 °C. Experiments were conducted in triplicate.

**Figure 10:**
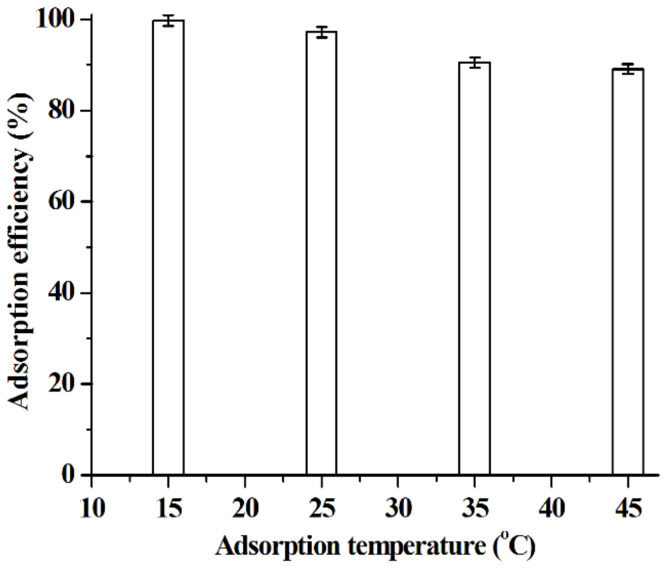
Effect of temperature on the adsorption efficiency of [C_3_mim]^+^Cl^−^@SiO_2_. Experimental conditions: C_baicalin_ = 5.0 × 10^−5^ mol·L^−1^; [C_3_mim]^+^Cl^−^@SiO_2_, 40 mg; volume of baicalin aqueous solution, 10 mL; adsorption time, 10 min; pH 5.0. Experiments were conducted in triplicate.

In this work, the parameters affecting the adsorption ability of [C_3_mim]^+^Cl^−^@SiO_2_ including adsorption time, pH and adsorption temperature were investigated. Data shown in Figure 8 indicate that the adsorption of baicalin can be completed in 10 min. Therefore, 10 min is selected as the optimal adsorption time. The experimental results illustrated in Figure 9 suggest that the adsorption efficiency increases with the pH increasing from 2.0 to 5.0 and then keeps constant with further increasing the pH values. It is known that the dissociation constant (p*K*_a_) of baicalin is 2.9 [46]. When pH > p*K*_a_, baicalin exists in the form of anion, meaning that there exists strong electrostatic attraction between baicalin anion and the cation of [C_3_mim]^+^Cl^−^@SiO_2_. That is to say, high adsorption efficiency would be achieved when pH > p*K*_a_; 5.0 is thus regarded as the optimal pH value. As shown in Figure 10, the adsorption ability of [C_3_mim]^+^Cl^−^@SiO_2_ decreases with the increase of temperature, indicating that the adsorption of baicalin is an exothermic adsorption process [47] and lower temperature is favorable for extraction. Thus, room temperature 25 °C is selected for the adsorption of baicalin.

### 2.5. Adsorption and Purification of Baicalin from the Root Extract of SBG

**Figure 11:**
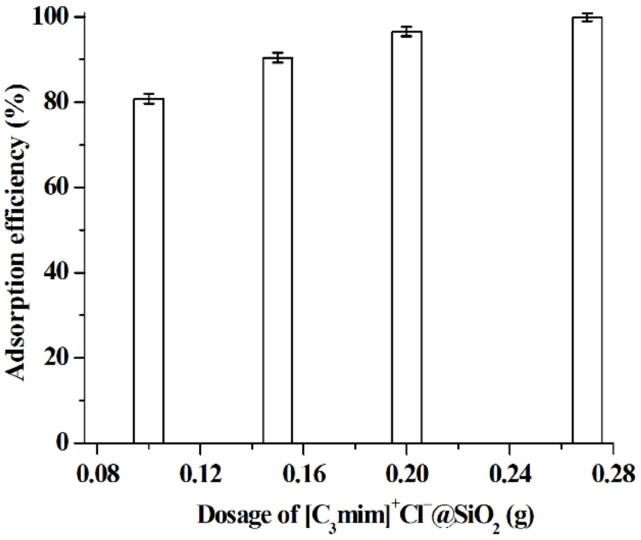
Effect of dosage of [C_3_mim]^+^Cl^−^@SiO_2_ on the adsorption of baicalin from 10 mL of the root extract of SBG. Experimental conditions: C_baicalin_ = 1.9 × 10^−3^ mol·L^−1^; adsorption time, 10 min; pH 5.0; adsorption temperature, 25 °C. Experiments were conducted in triplicate.

**Figure 12:**
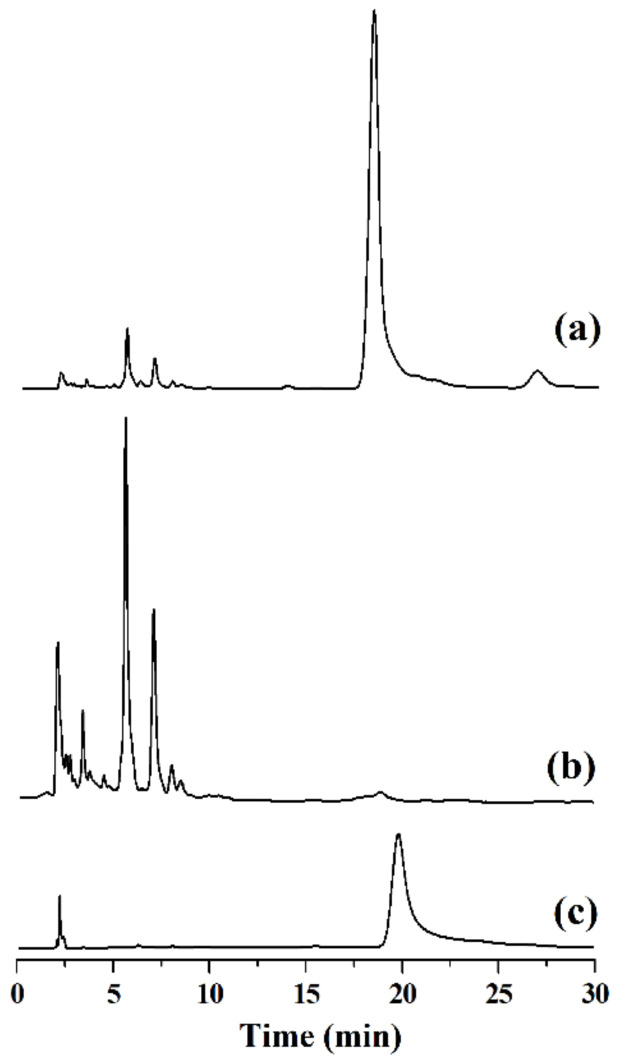
HPLC chromatograms of the root extract of SBG (**a**), the root extract of SBG after adsorption (**b**), and baicalin obtained from the desorption solution (**c**).

Under the selected adsorption conditions, [C_3_mim]^+^Cl^−^@SiO_2_ was used to absorb baicalin from the root extract of SBG. It is found that the baicalin concentration in the root extract of SBG is 1.9 × 10^−3^ mol·L^−1^. Data shown in Figure 11 indicate that 0.2 g of [C_3_mim]^+^Cl^−^@SiO_2_ can effectively adsorb baicalin from 10 mL of the extract (96.5%) and this adsorption condition is thus selected. To achieve the desorption of baicalin, [C_3_mim]^+^Cl^−^@SiO_2_ was washed with ethanol aqueous solution (50%, *v*/*v*, pH 3.0 adjusted by HCl) and the desorption efficiency is 97.4%. After removing most of the solvent by vacuum distillation, the baicalin powder crystallizes and its purity is 96.5% (determined by the aforementioned HPLC method). Typical HPLC chromatograms of baicalin before and after purification with [C_3_mim]^+^Cl^−^@SiO_2_ are shown in Figure 12. It can be seen that baicalin is successfully purified by [C_3_mim]^+^Cl^−^@SiO_2_.

### 2.6. Reusability of [C_3_mim]^+^Cl^−^@SiO_2_ and Comparison with Literature Methods

**Figure 13:**
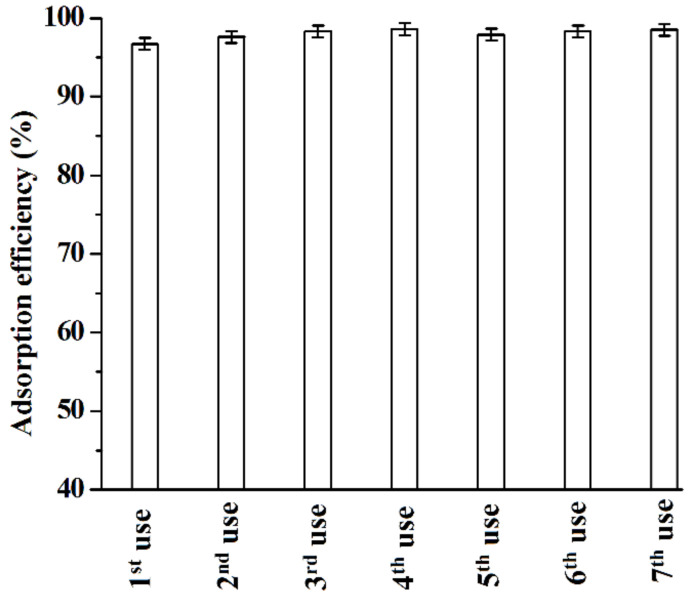
Reusability of [C_3_mim]^+^Cl^−^@SiO_2_ for the adsorption of baicalin from the root extract of SBG. Experimental conditions: adsorption temperature, 25 °C; adsorption time, 10 min; pH, 5.0; [C_3_mim]^+^Cl^−^@SiO_2_, 0.2 g; volume of the root extract of SBG, 10 mL. Experiments were conducted in triplicate.

In this work, the reusability of [C_3_mim]^+^Cl^−^@SiO_2_ is investigated and the results shown in Figure 13 indicate that [C_3_mim]^+^Cl^−^@SiO_2_ can be reused at least seven times without loss of its adsorption efficiency. Furthermore, as mentioned above, the HPD-100 macroporous resin and polyamide resin were used to adsorb and purify baicalin. Therefore, a comparison on the adsorption performance between [C_3_mim]^+^Cl^−^@SiO_2_ and the commercial adsorbents (HPD-100 macroporous resin and polyamide resin) was conducted and the results listed in Table 2 indicate that [C_3_mim]^+^Cl^−^@SiO_2_ has the strongest adsorption ability and the fastest adsorption rate and provides the highest purity compared with HPD-100 macroporous resin and polyamide resin.

## 3. Materials and Methods

### 3.1. Materials

Tetraethyl orthosilicate (TEOS, 98%) and (3-chloropropyl)trimethyoxysilane (CPTMOS, 97%) were obtained from Acros Organics (Bridgewater, NJ, USA). *N*-methylimidazole (MIM, 99%) and baicalin (98%) were supplied by Aladdin Bio-Chem Technology Co. (Shanghai, China). *N*-(2-hydroxyethyl)imidazole (C_2_OHIM, 98%) and *N*-benzylimidazole (BzIM, 98%) were purchased from Changzhou Chongkai Chemical Co., Ltd. (Changzhou, China). Roots of SBG were purchased from Hebeikangan Bio-Technology Co., Ltd. (Anguo, China) and ground into powder with a particle size of around 100 mesh. Macroporous resin (model HPD-100, 60–16 mesh) was obtained from Donghong Chemical Co., Ltd. (Kunshan, China). 1-Butyl-3-methylimidazolium bis(trifluoromethylsulfonyl)imide ([C_4_mim]NTf_2_) was purchased from Lanzhou Institute of Chemical Physics (Lanzhou, China).

### 3.2. Methods

#### 3.2.1. Synthesis of ILs Grafted Silica

Typically, 1.0 g of TEOS was dissolved into 1.0 g of [C_4_mim]NTf_2_, followed by the addition of 1.5 mL of ammonia solution (4.2 wt%). This mixture was stirred at room temperature for 20 min to produce silica gel and then 0.95 g of CPTMOS was added. The resultant mixture was subject to another 3 h of stirring. After dissolving with ethanol and filtering, CPTMOS-modified silica (denoted as C_3_Cl@SiO_2_) was obtained. To graft *N*-methylimidazolium-based IL on the surface of silica, CPTMOS-modified silica (0.2 g) and *N*-methylimidazole (0.33 g) were dispersed into 15 mL of ethanol. This mixture was stirred at 80 °C for 12 h to generate IL-modified silica (denoted as [C_3_mim]^+^Cl^−^@SiO_2_). The synthesized [C_3_mim]^+^Cl^−^@SiO_2_ was washed with ethanol and then dried at 60 °C for 3 h. The grafting of *N*-(2-hydroxyethyl)imidazolium- and *N*-benzylimidazolium-based ILs on the surface of silica followed a similar way and the resultant products were denoted as [C_3_C_2_OHim]^+^Cl^−^@SiO_2_ and [C_3_Bzim]^+^Cl^−^@SiO_2_, respectively.

#### 3.2.2. Characterization of ILs Grafted Silica

All the ILs grafted silica products were characterized by an elemental analyzer (model FLASH 2000, Thermo Fisher Scientific, Belmont, MA, USA), a field-emission scanning electron microscope (FE-SEM, Quanta 250 FEG, Thermo Fisher Scientific, Hillsboro, OR, USA), a transmission electron microscope (TEM, model Tecnai G2 20, FEI, Hillsboro, OR, USA), a surface area and porosity analyzer (model ASAP 2460, Micromeritics Instrument Corp., Norcross, GA, USA), a Fourier transform infrared (FT-IR) spectrophotometer (model V70, Bruker Optic GmbH, Ettlingen, Germany) and a laser particle sizer (model Mastersizer 2000, Malvern Instruments Ltd., Malvern, UK). The FT-IR spectra of the ILs grafted silica in transmission mode were measured by the KBr pressed disc method. The particle size distribution of the ILs grafted silica was determined by the laser diffraction method using Mastersizer 2000 and water was used as dispersive solvent.

#### 3.2.3. Preparation of the Root Extract of SBG

The preparation of the root extract of SBG was conducted by referring to the reported work with minor modification [12,15]: 1.0 g of SBG powder and 100 mL of water were mixed under stirring at 100 °C for 30 min. After filtering, the water phase was collected and stored in refrigerator before use.

#### 3.2.4. Determination of Baicalin

The baicalin concentration in water phase was determined by high performance liquid chromatography (HPLC) using an Agilent 1200 HPLC system (Agilent Technologies, Santa Clara, CA, USA). The chromatographic conditions are as follows: mobile phase, the mixture of acetonitrile (17%, *v*/*v*) and 0.1% (*v*/*v*) of acetic acid aqueous solution (83%, *v*/*v*); flow-rate, 0.8 mL·min^−1^; separation column, ZORBAX Eclipse XDB-C18 column (4.6 × 150 mm, 5 μm, Agilent); column temperature, 30 °C; detection wavelength, 275 nm; injection volume, 5 μL.

#### 3.2.5. Adsorption and Desorption of Baicalin

For a typical adsorption procedure, 40 mg of [C_3_mim]^+^Cl^−^@SiO_2_ was mixed with 10 mL of baicalin aqueous solution (5.0 × 10^−5^ mol·L^−1^, pH 5.0) under stirring at room temperature for 10 min. After filtering, [C_3_mim]^+^Cl^–^@SiO_2_ powder was washed with ethanol aqueous solution (50%, *v*/*v*, pH 3.0 adjusted by HCl) to recover baicalin and to regenerate the absorbent.

The adsorption capacity (*Q*_e_) and adsorption efficiency (*E*) are expressed by the following equations (Equations (1) and (2)):

(1) $$Q_{e}=\frac{V(C_{0}-C_{e})}{m}$$

(2) $$E=\frac{\left(C_{0}-C_{e}\right)}{C_{0}}\times 100\%$$

where *Q*_e_ (mg·g^−1^ dry absorbent), *V*, *C*_0_, *C*_e_, *m*, *E*, are the adsorption capacity of absorbent to baicalin at equilibrium, volume of baicalin solution, initial baicalin concentration, equilibrium concentration of baicalin after adsorption, dry weight of adsorbent and adsorption efficiency, respectively.

The adsorption behavior of adsorbent was analyzed using Langmuir, Freundlich, and Dubinin–Radushkevich equations [15,42,43,44,45], respectively:

Langmuir equation (Equation (3)):

(3) $$\frac{1}{Q_{e}}=\frac{1}{Q_{m}K_{L}}\frac{1}{C_{e}}+\frac{1}{Q_{m}}$$

Freundlich equation (Equation (4)):

(4) $$\ln Q_{e}=\ln K_{F}+\frac{1}{n}\ln C_{e}$$

where *Q*_e_, *Q*_m_, *K*_L_, *C*_e_, *K*_F_, and 1/*n* are the adsorption capacity (mg·g^−1^ dry absorbent), the maximum adsorption capacity (mg·g^−1^ dry absorbent), the Langmuir constant, the equilibrium concentration of baicalin after adsorption, the Freundlich constant and an empirical constant related to the adsorption intensity, respectively.

The Dubinin–Radushkevich equations are expressed as (Equations (5)–(7)):

(5) $$\ln Q_{e}=\ln Qm-K\varepsilon^{2}$$

(6) $$\varepsilon =RT\ln(1+1/C_{e})$$

(7) $$E=1/\sqrt{2K}$$

where *K* (mol^2^·kJ^−2^) is the activity coefficient related to mean free energy of adsorption; *ε* (kJ·mol^−1^) is the Polanyi potential; R (8.314 J·mol^−1^·K^−1^) is the gas constant and *T* (K) is Kelvin temperature; *E* (kJ·mol^−1^) is the average free energy of adsorption.

## 4. Conclusions

In this work, a two-step method was developed to prepare ILs grafted silica: (I) the co-hydrolysis and co-condensation of CPTMOS and TEOS in [C_4_mim]NTf_2_ medium to produce C_3_Cl@SiO_2_; and (II) the quaternization reaction between C_3_Cl@SiO_2_ and imidazole derivatives to generate ILs grafted silica. Compared with the reported methods, the developed technique to prepare ILs grafted silica was easier to operate and more environmentally benign because the use of toxic solvents, such as dimethylformamide and toluene was avoided. Compared with the commercial HPD-100 macroporous resin and polyamide resin, the synthesized IL grafted silica, [C_3_mim]^+^Cl^−^@SiO_2_ exhibited strongest adsorption ability, fastest adsorption rate and could provide highest purity of baicalin (96.5%). These results suggested that ILs were promising media for the preparation of silica-based materials and had huge potential in the synthesis of other functional materials. Finally, ILs grafted silica exhibited great potential in the separation and purification of baicalin from SBG and may be applied for the adsorption and purification of other bioactive compounds with similar chemical structures.

## Figures and Tables

**Table 1:** Isotherm model parameters for the adsorption of baicalin onto ILs grafted silica (pH 5.0, 25 °C).

Isothermal Absorption Models	Parameters	[C_3_mim]^+^Cl^−^@SiO_2_	[C_3_C_2_OHim]^+^Cl^−^@SiO_2_	[C_3_Bzim]^+^Cl^−^@SiO_2_
Langmuir model	Fitted equation	1/*Q*_e_ = 0.0039/*C*_e_ + 0.0028	1/*Q*_e_ = 0.0035/*C*_e_ + 0.0034	1/*Q*_e_ = 0.0037/*C*_e_ + 0.0038
	Correlation coefficient (*r*)	0.9991	0.9981	0.9920
	*Q*_m_ (mg·g^−1^)	357.1	294.1	263.2
Freundlich model	Fitted equation	ln*Q*_e_ = 0.3679ln*C*_e_ + 5.0774	ln*Q*_e_ = 0.3264ln*C*_e_ + 5.0144	ln*Q*_e_ = 0.2959ln*C*_e_ + 4.9458
	*r*	0.9929	0.9841	0.9990
	1/*n*	0.3679	0.3264	0.2959
	*K*_F_ (mg·g^−1^)	160.4	150.6	140.6
Dubinin–Radushkevich model	Fitted equation	ln*Q*_e_ = −0.0977*ε*^2^ + 5.5808	ln*Q*_e_ = −0.0837*ε*^2^ + 5.4671	ln*Q*_e_ = −0.0846*ε*^2^ + 5.3615
	*r*	0.9741	0.9833	0.9622
	*E* (kJ·mol^−1^)	2.26	2.44	2.43
	*Q*_m_ (mg·g^−1^)	265.3	236.8	213.0

**Table 2:** Comparison on the adsorption performance between [C_3_mim]^+^Cl^–^@SiO_2_ and commercial adsorbents.

Adsorbent	*Q*_e_ (mg·g^−1^)	Adsorption Time (min)	Purity of Baicalin After Desorption
[C_3_mim]^+^Cl^−^@SiO_2_ (this work)	357.1	10	96.5%
HPD-100 macroporous resin [15]	178.57	180	58.3%
Polyamide resin [13]	233.23	20	33.86%

## Data Availability

Data is contained within the article.

## Supplementary Materials

The following are available online. Figure S1: The FE-SEM (A) and TEM (B) images of [C_3_C_2_OHim]^+^Cl^−^@SiO_2_; Figure S2. The FE-SEM (A) and TEM (B) images of [C_3_Bzim]^+^Cl^–^@SiO_2_; Figure S3. Particle size distribution of [C_3_C_2_OHim]^+^Cl^–^@SiO_2_; Figure S4. Particle size distribution of [C3Bzim]^+^Cl^–^@SiO_2_.

## Sample Availability

Samples of the compounds baicalin and ILs grafted silica are available from the authors.
