# Supplementary material for: Adsorption and Purification of Baicalin from Scutellaria baicalensis Georgi Extract by Ionic Liquids (ILs) Grafted Silica
Source: Molecules. 2021 Apr 16;26(8):2322. doi: 10.3390/molecules26082322 (PMC8073518; doi:10.3390/molecules26082322)
Supplement: Supplementary file 1 [file molecules-26-02322-s001.zip › molecules-1169625-supplementary.pdf]

molecules

MDPI

Supplementary Materials

Adsorption and Purification of Baicalin from *Scutellaria Baicalensis* Georgi Extract by Ionic Liquids (ILs) Grafted Silica

Yunchang Fan[1,*], Di Wu[1] and Sheli Zhang[2]

[1] College of Chemistry and Chemical Engineering, Henan Polytechnic University, Jiaozuo 454003, China
[2] College of Science and Technology, Jiaozuo Teachers College, Jiaozuo, 454000, China
* Correspondence: yunchangfan2009@163.com; Tel.: +863913986813, Fax: +863913987815.

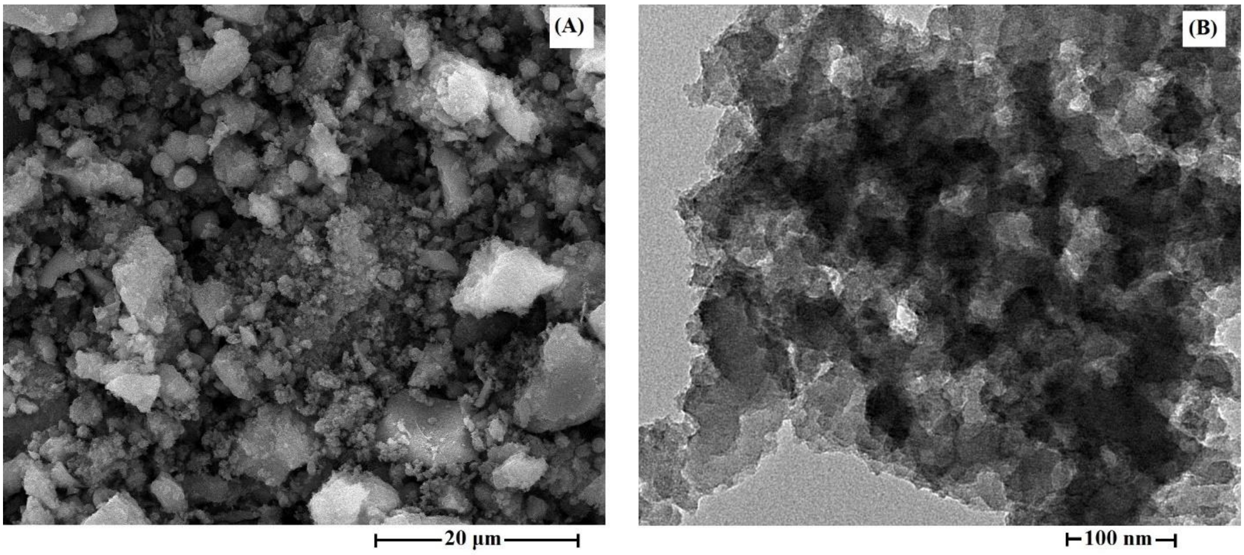

Figure S1. The FE-SEM (**A**) and TEM (**B**) images of $[C_3C_2OHim]^+Cl^-@SiO_2$.

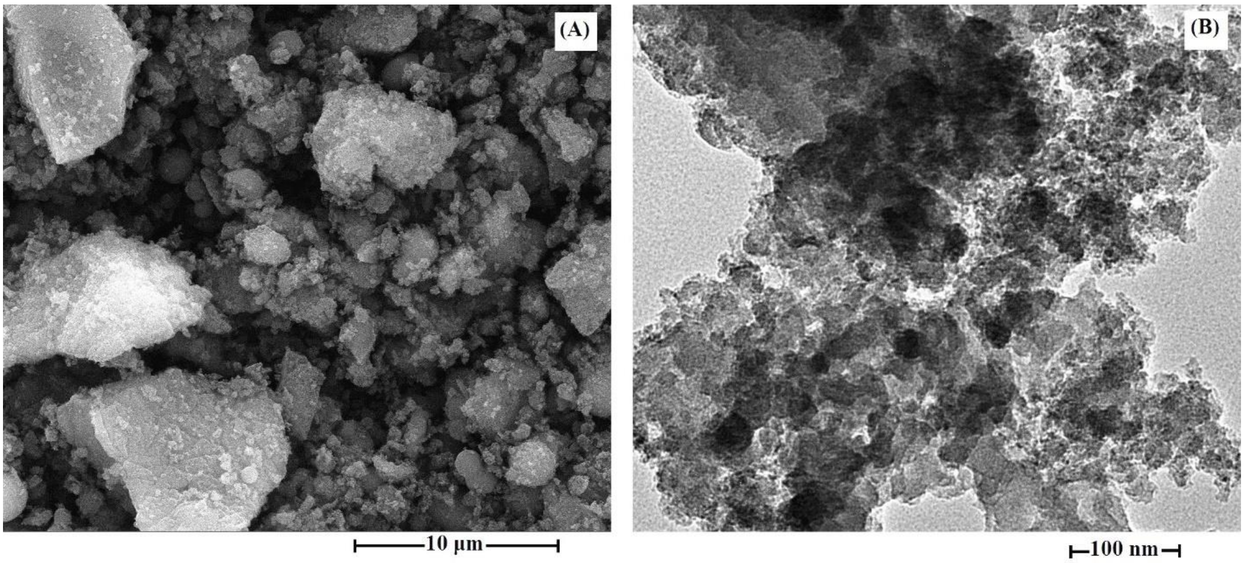

Figure S2. The FE-SEM (**A**) and TEM (**B**) images of $[C_3Bzim]^+Cl^-@SiO_2$.

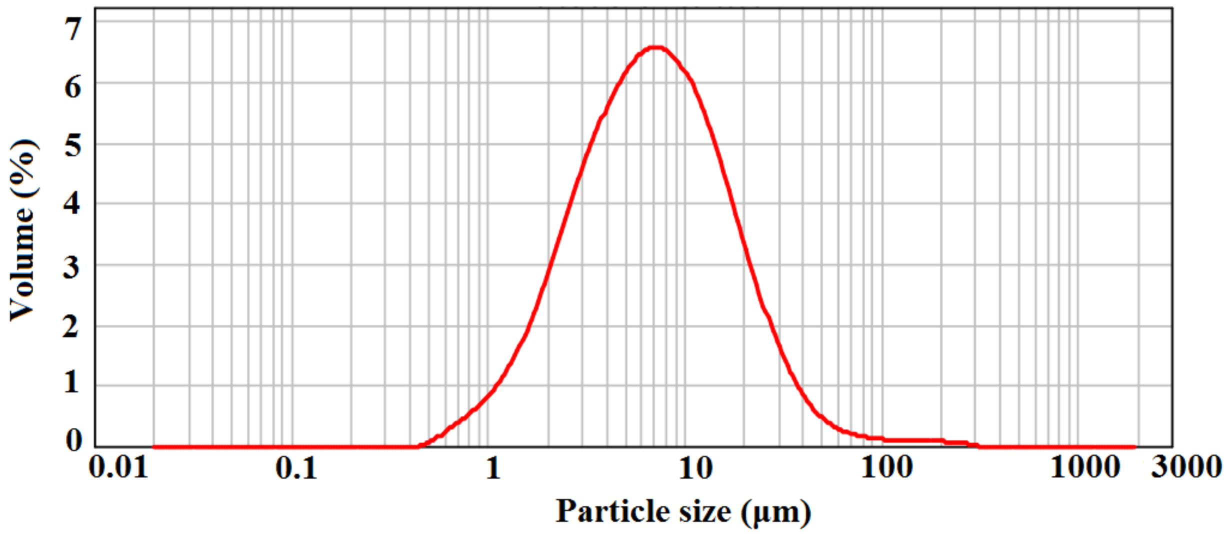

Figure S3. Particle size distribution of $[C_3C_2OHim]^+Cl^-@SiO_2$.

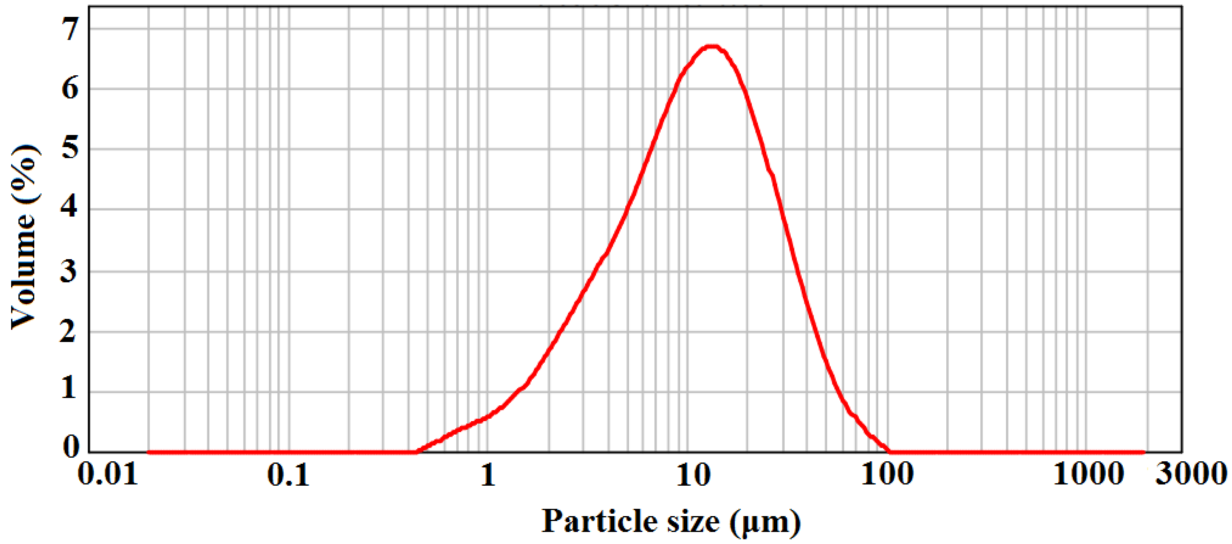

Figure S4. Particle size distribution of $[C_3Bzim]^+Cl^-@SiO_2$.
